# Retraction: Experimental and theoretical investigations of Erbium complex: DNA/BSA interaction, anticancer and antibacterial studies

**DOI:** 10.3389/fchem.2024.1433689

**Published:** 2024-05-24

**Authors:** Frontiers Editorial Office

Following publication, concerns were raised regarding the validity of the data in the article.

Following provision of raw data by the authors, the Chief Editor concluded that the article’s conclusions and assertions were not sufficiently supported by the findings from the material provided; therefore, the article has been retracted.

This retraction was approved by the Chief Editors of Frontiers in Chemistry and the Chief Executive Editor of Frontiers.

The authors have not agreed to this retraction.

